# Exosomal ACADM sensitizes gemcitabine-resistance through modulating fatty acid metabolism and ferroptosis in pancreatic cancer

**DOI:** 10.1186/s12885-023-11239-w

**Published:** 2023-08-23

**Authors:** Yuhan Yang, Haitao Gu, Kundong Zhang, Zengya Guo, Xiaofeng Wang, Qingyun Wei, Ling Weng, Xuan Han, Yan Lv, Meng Cao, Peng Cao, Chen Huang, Zhengjun Qiu

**Affiliations:** 1 Department of General Surgery, Shanghai General Hospital, Shanghai Jiao Tong University School of Medicine, Shanghai, 200080 China; 2 Affiliated Hospital of Integrated Traditional Chinese and Western Medicine, Nanjing University of Chinese Medicine, 100 Hongshan Road, Nanjing, 210028 Jiangsu China; 3 Department of Pharmacology, School of Pharmacy, Nanjing University of Chinese Medicine, Nanjing, 210023 China; 4 Department of Thoracic Surgery, Ruijin Hospital, Shanghai Jiao Tong University School of Medicine, Shanghai, 200025 China

**Keywords:** Exosomes, Lipid metabolism, Ferroptosis, Pancreatic cancer, Biomarker, Gemcitabine

## Abstract

This study aimed to evaluate the potential of exosomes from cancer cells to predict chemoresistance in pancreatic cancer (PC) and explore the molecular mechanisms through RNA-sequencing and mass spectrometry. We sought to understand the connection between the exosomal Medium-chain acyl-CoA dehydrogenase (ACADM) level and the reaction to gemcitabine in vivo and in patients with PC. We employed loss-of-function, gain-of-function, metabolome mass spectrometry, and xenograft models to investigate the effect of exosomal ACADM in chemoresistance in PC. Our results showed that the molecules involved in lipid metabolism in exosomes vary between PC cells with different gemcitabine sensitivity. Exosomal ACADM (Exo-ACADM) was strongly correlated with gemcitabine sensitivity in vivo, which can be used as a predictor for postoperative gemcitabine chemosensitivity in pancreatic patients. Moreover, ACADM was found to regulate the gemcitabine response by affecting ferroptosis through Glutathione peroxidase 4 (GPX4) and mevalonate pathways. It was also observed that ACADM increased the consumption of unsaturated fatty acids and decreased intracellular lipid peroxides and reactive oxygen species (ROS) levels. In conclusion, this research suggests that Exo-ACADM may be a viable biomarker for predicting the responsiveness of patients to chemotherapy.

**Supplementary Information:**

The online version contains supplementary material available at 10.1186/s12885-023-11239-w.

## Introduction

Despite pancreatic cancer (PC)’s low incidence rate, it has the highest mortality rate among all major cancers and is projected to become the second most fatal form of cancer by 2030 [1]. Surgery is the most effective treatment for PC. Yet, many patients are not diagnosed in time and miss the opportunity for operative treatment. PC can spread to other body parts, and micrometastases, which cannot be detected during surgery, could cause a tumor recurrence after the procedure. Therefore, patients with PC usually require systemic chemotherapy in addition to surgery. Adjuvant therapy was performed using a modified FOLFIRINOX regimen and gemcitabine monotherapy or combination therapy is the typical first-line chemotherapy regimen for PC [2]. FOLFIRINOX is more effective than gemcitabine, however the latter is still the first line of systemic chemotherapy due to its lower bone marrow suppression side effect [3]. Despite the improved prognosis for those receiving systemic chemotherapy after surgery, 40% of patients still relapse within one year after the procedure [4]. As there are no effective imaging or blood markers, it is necessary to explore chemoresistance characteristics to optimize an effective or optimal PC therapy as blood markers are specific to chemotherapy sensitivity.

Exosomes, small vesicles measuring between 30 and 150 nm in diameter and released by various cells, are believed to facilitate communication and material transfer between cells [5]. Recent studies have demonstrated that exosomes derived from PC can induce macrophage polarization and angiogenesis [6]. Additionally, exosomes taken up by chemotherapy-resistant breast cancer cells have been found to activate the NOTCH, and NT stem cell pathways by targeting NUMB and DKK3, respectively, resulting in the emergence of drug resistance [7]. The potential of exosomes as markers for PC diagnosis is currently being explored, as liquid biopsy is a rapid and convenient method of diagnosis, even in cases where pre-operative biopsy is challenging to perform [8]. Research has demonstrated that the exosomes’ substances differ between healthy and cancerous patients, and thus, detecting exosomal markers may significantly improve the accuracy of a cancer diagnosis [9].

The regulation of fatty acid decomposition and anabolism is indispensable for maintaining intracellular energy levels. High rates of fatty acid oxidation are necessary for the accelerated growth of tumors, their sensitivity to treatment, and their capacity to proliferate and metastasize. For instance, an increase in fatty acid synthase (FASN) can increase resistance to cisplatin in breast and ovarian cancer [10]. The phosphorylation of acetyl-CoA carboxylase (ACC) is associated with a greater sensitivity to cetuximab treatment in head and neck cancer, as well as lung cancer [11].

Studies have demonstrated that the overproduction of reactive oxygen species (ROS) can be cytotoxic and lead to cell death [12]. However, research has demonstrated that ferroptosis and anti-ROS abnormality in chemotherapy-resistant cells are associated with survival [13, 14]. Glutathione peroxidase 4 (GPX4) has been identified as a potential target to reverse chemoresistance in PC, [15] due to its ability to convert lipid peroxides into non-toxic fatty alcohols and protect cells from their damage [16]. However, the way tumor cells with an excessive fatty acid metabolism control GPX4 to avoid ferroptosis caused by lipid peroxidation has yet to be determined.

This study demonstrated the potential of exo-Medium-chain acyl-CoA dehydrogenase (ACADM) to predict gemcitabine sensitivity in both in vitro and *in vivo.* As such, exo-ACADM has the potential to be a reliable indicator for gauging the effectiveness of chemotherapy treatments.

## Materials and methods

### Cell culture

PC cell lines SW1990, and Miapaca2 were obtained from the Department of Pathophysiology, Key Laboratory of Cell Differentiation and Apoptosis of the National Ministry of Education (Shanghai Jiao Tong University School of Medicine, Shanghai, 200,025, China). The remaining cell lines (including PANC1, Bxpc-3, Aspc-1, su86.86, CFPAC, and HPAC) were obtained from ATCC (Manassas, VA). Short tandem repeat (STR) DNA was used to identify the above cell lines. All mycoplasma tests represented negative outcomes. Cell lines were cultured in RPMI 1640 medium supplemented with 0.10% fetal calf serum, 1% penicillin, and streptomycin under a 5% carbon dioxide atmosphere at 37℃ .

### Exosome collection, isolation, and purification

PC cells were initially implanted into T75 culture flasks in a quantity of 5 × 10^6^. To collect exosomes, 10% exosome-free FBS 1640 was applied, and ultracentrifugation was employed. The process of ultracentrifugation began by removing large vesicles and cell fragments from the supernatant via centrifugation at 300 g for 5 min, 2,000 g for 5 min, and 12,000 g for 30 min, followed by filtration using a 0.22-mm sieve. The exosomes were then isolated by ultracentrifugation at 110,000 g with a washing process using PBS suspension, and the suspension was stored in 200uL of PBS buffer. A precipitation method using ExoJuice (ExonanoRNA, Foshan, People’s Republic of China) was employed per the manufacturer’s instructions for isolating exosomes in human blood samples. The procedure began with collecting cell culture via centrifugation at 12,000 g for 30 min. Subsequently, the supernatant was transferred to an ultracentrifuge tube, and 1mL of ExoJuice was added to the bottom of the centrifuge tube. After 70 min of centrifugation at 100,000 g, the first 500 mL of liquid from the bottom was discarded. The remaining 300mL of fluid containing the purified exosomes was carefully collected and preserved.

### RNA-sequencing

Total RNA was extracted from samples using a Trizol reagent kit (Invitrogen, Carlsbad, CA, USA) according to the manufacturer’s instructions. RNA quality was evaluated using Agilent 2100 Bioanalyzer (Agilent Technologies, Palo Alto, CA, USA) and verified by RNase-free agarose gel electrophoresis. Subsequently, eukaryotic mRNA was enriched by Oligo (dT) beads. Fragmentation of the enriched mRNA was done with fragmentation buffer, and reverse transcription into cDNA was performed using NEBNext Ultra RNA Library Prep Kit for Illumina (NEB #7530, New England Biolabs, Ipswich, MA, USA). The purified double-stranded cDNA fragments were end-repaired, and A base was added and ligated to Illumina sequencing adapters. The ligation reaction was purified with the AMPure XP Beads (1.0X). The ligated fragment size was selected by agarose gel electrophoresis, and polymerase chain reaction (PCR) amplification was performed. The resulting cDNA library was sequenced using Illumina Novaseq6000 by Gene Denovo Biotechnology Co.

### Mass spectrometry

Exosomes of each PC cells were transferred into lysis buffer (2% SDS, 7 M urea, 1 mg/ml protease inhibitor cocktail) and homogenized at four °C using an ultrasonic homogenizer for 3 min (for bacteria, 5 min; for tissue, 180 s three times). The homogenate was centrifuged at 15,000 rpm for 15 min, and the supernatant was collected for protein concentration estimation using the BCA Protein Assay Kit. Subsequently, 50 µg proteins were suspended in 50µL solution and reduced by adding 1µL of 1 M dithiotreitol at 55 °C for 1 h. The sample was then alkylated with 5µL of 20mM iodoacetamide in the dark at 37 °C for 1 h. After this, the sample was precipitated with 300µL prechilled acetone at -20 °C overnight. The precipitate was washed twice with cold acetone and re-suspended in 50 mM ammonium bicarbonate. Finally, the proteins were digested with sequence-grade modified trypsin (Promega, Madison, WI) at a substrate/enzyme ratio of 50:1 (w/w) at 37 °C for 16 h. Mass spectrometry was then performed under data-dependent acquisition mode, where automatic switching between MS and MS/MS modes was enabled.

### Nanoparticle tracking analysis (NTA)

Firstly, 10µL of exosomes from each cell line were mixed evenly into 1mL of PBS. The particle size analyzer was then cleaned with sterile PBS after 0.22 μm biofilm filtration to remove any potential interference from impurities. Subsequently, the exosome solution was drawn up with a 1mL syringe and dripped into the analyzer from the upper sample tube. Finally, the particle size analysis software was opened, and a dynamic nano video was recorded to capture the nano particles and analyze the count. To ensure accuracy, care was taken to exclude any impurities that may have been present during the filming.

### Drug treatment studies

Gemcitabine, sourced from Sigma (St. Louis, MO), was diluted with PBS to eight different concentrations and then seeded into 96-well plates at 1 × 10^3^ cells per well after cell adherence and incubated for one day. Subsequently, the cell viability was evaluated using Cell-Counting-Kit-8 (Yeason, China, 40203ES60), and the IC50 values were calculated using GraphPad Prism 6 (GraphPad Software, San Diego, CA). According to the previously reported criteria (Insert citation DIO: 1158/1078 − 0432.CCR-20-2475), PC cell lines were divided into the gemcitabine-sensitive group and the gemcitabine-insensitive group.

### Cell transfection

293T cells and a lentiviral packaging kit (Yeason, China) were used for packaging the ACADM knockdown plasmid and overexpression plasmid into the lentivirus. Subsequently, PC cells were transfected and exposed to puromycin (4 ug/ml) for seven days, and single clones were selected using the limited dilution method for further experiments. Lipo3000 was used to perform transient transfection and transfection of 293T cells. The instructions were followed to prepare liquid A and liquid B, which were incubated at room temperature for 10 minutes before adding Optim medium. The cells were cultured under standard conditions for 24 hours before changing the medium. The si-ACADM sequences were as follows: si1: 5’-CCTGAGAAGTATTTCTCGTTT-3’; si2: 5’-GTGCAGATACTTGGAGGCAAT-3’.

### Patient samples

The Medical Ethics Committee approved this study, and Animal Use and Shanghai General Hospital provided the Management Committee of Shanghai General Hospital IRB approval (No. 2021SQ099). Each patient gave written consent. A total of 71 postoperative PDAC specimens that had been treated with gemcitabine chemotherapy were collected, and the patients were monitored over time.

### Fatty acid oxidation (FAO) measurements

For FAO assays, 2 × 10^6^ cells were seeded into 6-well plates and incubated for 24 h prior to treatment with gemcitabine for 48 h. Subsequently, a Lipid Peroxidation (MDA) Assay kit (ab118970, Abcam, UK) was used to test the cells. The cells were homogenized in Lysis Solution (Buffer + BHT) using a Dounce homogenizer on ice and centrifuged at 12,000 x g for 10 min to eliminate insoluble materials. Finally, a microplate reader was used to measure the absorbance at 532 nm OD.

### In vivo xenografts model

The Ethics Committee for Animal Research of Shanghai Jiaotong University School of Medicine (Shanghai, People’s Republic of China) approved all animal experiments. As reported in prior studies, PC cells (Bxpc-3, PANC1, CFPAC, miapaca-2, SW1990, HPAC, su86.86, and Aspc-1) were implanted subcutaneously into the right flank of 4-week-old BALB/c male nude mice. The size of the tumor was measured every three days using a digital caliper. When the tumor volume was approximately 100 mm [2] on the sixth day, the mice were randomly divided into sensitive and insensitive groups, and gemcitabine was intraperitoneally administered every three days. After five injections, the mice killed for cervical dislocation after carbon dioxide anesthesia were sacrificed to remove the primary tumors and record their weights.

### Statistical analysis

All data were expressed as mean ± standard deviation (SD). Statistical analysis was performed with Graphpad Prism 9. The student’s t-test and the analysis of variance (ANOVA) were utilized to contrast continuous variables.

## Results

### Distinct exosomal gene expression profiles in PC cells with different sensitivities for gemcitabine

**Fig. 1:**
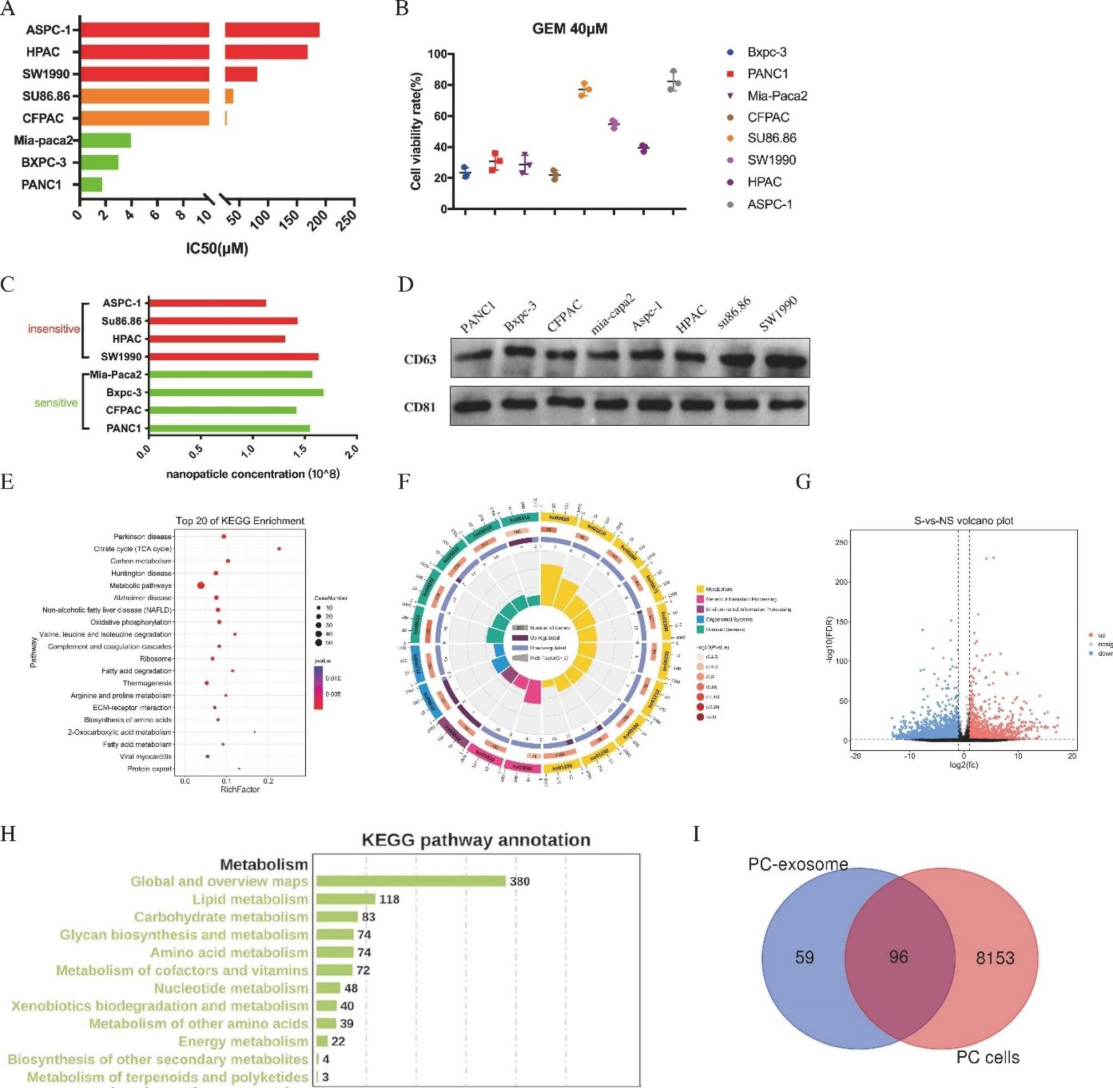
Distinct exosomal gene expression profiles in pancreatic cancer cells with different sensitivity [17]. **(A)** Median inhibitory dose of gemcitabine in different PC cells (IC50). **(B)** Cell viability of PC cells under 40 μm of gemcitabine. **(C)** Nanoparticle concentration of exosomes from pancreatic cancer cells detected by NTA. **(D)** The total amount of exosomes in pancreatic cancer cells was detected by WB. **(E and F)** KEGG pathway analysis of mass spectrometry results of exosomes proteins between sensitive and insensitive PC cells. **(G)** The volcano map reflects genes difference between sensitive and insensitive pancreatic cancer cells. **(H)** KEGG pathway analysis of RNA-seq results between sensitive and insensitive PC cells. **(I)** Wayne diagram shows the intersection of differential molecules of PC-derived exosomes and PC cells

An analysis was conducted to examine the sensitivity of eight PC cell lines to GEM, with the IC50 concentrations being determined (Fig. 1A). The results showed that four cell lines (Bxpc-3, PANC1, Miapaca-2 and CFPAC) had IC50 values of 1.7µM, 3.0µM, 3.9µM and 27.94µM, respectively, while the remaining four cell lines (su86.86, SW1990, HAPC and Aspc-1) had IC50 values of 39.6µM, 81.0µM, 169.0µM and 189.8µM, respectively. Consequently, the cell lines were divided into two groups: the gemcitabine-sensitive group (Miapaca-2, Bxpc-3, CFPAC, and PANC1) and the gemcitabine-insensitive group (Aspc-1, su86.86, HAPC and SW1990) (Fig. 1B). Subsequently, the number of exosomes produced by the PC cells with varying sensitivities was compared. Exosomes were extracted from each cell line by ultracentrifugation and were identified using NTA and western blotting, including CD63 and CD81. The results showed no variation in the number of exosomes among the cell lines within 48 h (Fig. 1C). Additionally, western blotting of exosome markers revealed no significant difference between the cell lines (Fig. 1D). NTA and mass spectrometry analysis revealed 155 molecules that were differentially expressed between the sensitive and insensitive cell lines, which were significantly enriched in metabolic pathways such as the TCA cycle, oxidative phosphorylation, and fatty acid metabolism (Fig. 1E-F). To further investigate the molecular differences between the two groups, RNA-seq was conducted, resulting in the identification of 8249 differentially expressed genes (Fig. 1G). Interestingly, intracellular molecules were abundant in the lipid metabolic pathway (Fig. 1H). We conducted further analysis to explore the common molecules between RNA-seq and exosome mass spectrometry and their functions (Fig. 1I). Our findings suggest that there are notable discrepancies in metabolic-related substances among exosomes of PC cells with different chemosensitivity.

### Exosomal ACADM predicted gemcitabine sensitivity of PC in vitro and in vivo

**Fig. 2:**
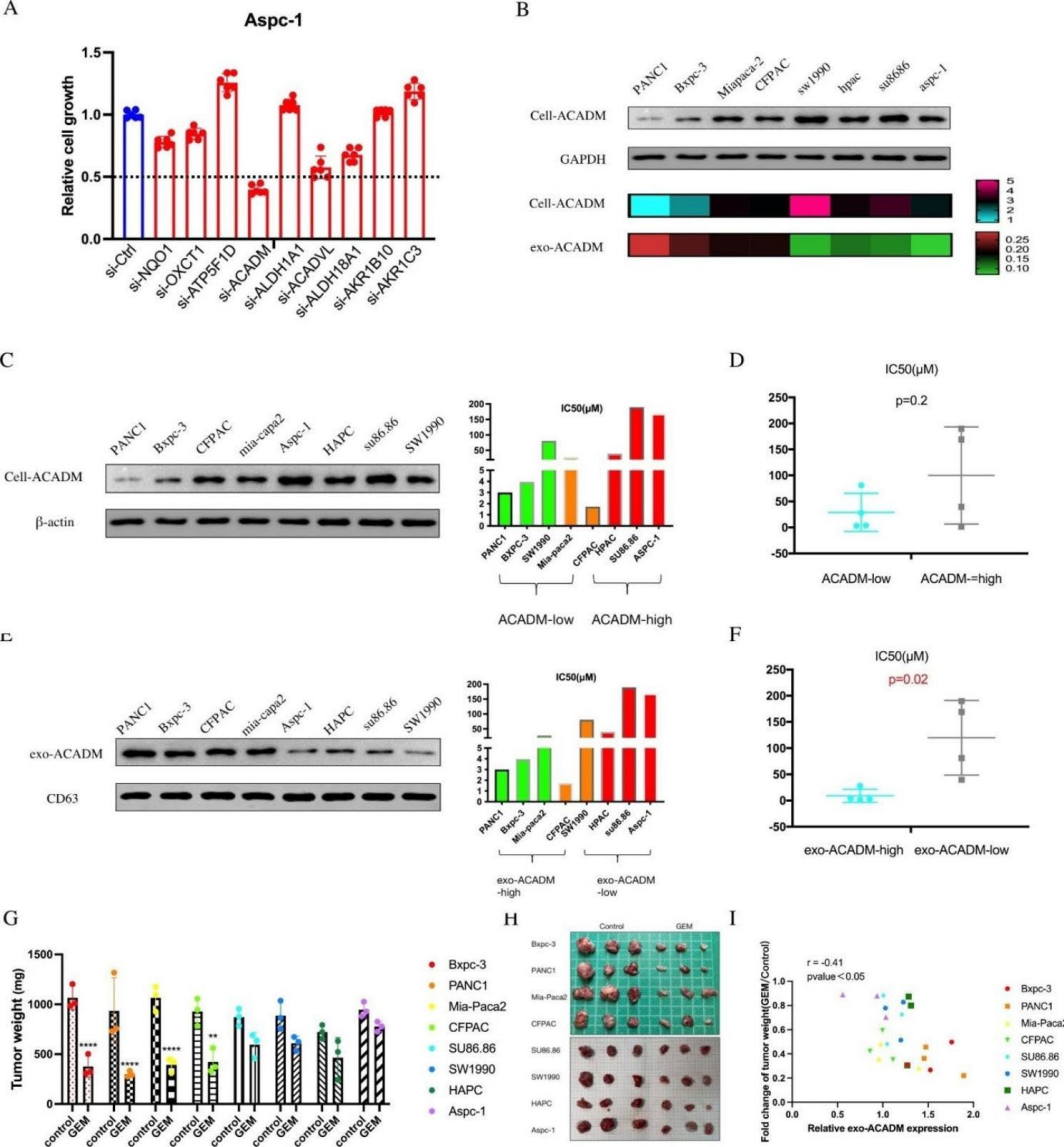
Exosomal ACADM predicted gemcitabine sensitivity of pancreatic cancer in vitro and in vivo. **(A)** CCK-8 assays indicated the cell growth of cells transfected with each siRNA. **(B)** The ACADM level in PC cells and its exosomes were detected by WB. **(C)** The ACADM level of each PC cell line was detected by WB. **(D)** IC50 values calculated from dose-response curves of high and low ACADM groups. **(E)** The ACADM level of PC cell-derived exosomes was detected by WB. **(F)** IC50 values calculated from dose-response curves of high and low exo-ACADM groups. **(G)** Tumor weight of xenografts treated or not with gemcitabine. **(H)** Xenografts of each cell line (gemcitabine-treated or control). **(I)** Correlation analysis between differential multiples of tumor weight and exo-ACADM in blood after gemcitabine treatment

RNA-seq analysis and mass spectrometry of exosome of PC cells with varying chemosensitivity revealed that metabolic processes might be a significant factor in determining sensitivity. To further investigate, we generated siRNAs for several differentially expressed metabolic-related genes and conducted CCK-8 assays. The results showed that when compared to the control group, cell viability increased after knocking down ATP5F1D, ALDH1A1, AKR1B10, and AKR1C3, while cell viability decreased after knocking down NQO1, OXCT1, ACADM, ACADVL, and ALDH18A1 (Fig. 2A). Notably, the chemosensitivity of PC cells was improved after ACADM knockdown.

Then, western blotting assay was used to detect the levels of ACADM in cells and exosomes, which revealed that cells with high expression of ACADM had lower ACADM content in the exosomes (Fig. 2B). To further evaluate the potential of ACADM as a biomarker in exosomes, the relationship between ACADM and gemcitabine sensitivity in PC cell lines and their exosomes was analyzed. Although the expression of ACADM in the cell lines was not significantly associated with their sensitivity to gemcitabine (Fig. 2C and D), the expression of ACADM in the exosomes was correlated considerably with gemcitabine sensitivity (Fig. 2E F). Additionally, the tumor weight of mice injected with gemcitabine-sensitive cells was lower than that of the control group, while the tumor weight of mice injected with gemcitabine-insensitive cells was reversed (Fig. 2G-H). Moreover, a significant negative correlation was observed between exo-ACADM and tumor weight (r=-0.41) after gemcitabine treatment (Fig. 2I). These experiments demonstrated that exosomal ACADM could predict the sensitivity of PC to gemcitabine treatment in vivo and in vitro.

### Predictive value of exo-ACADM for chemotherapy relapse

**Fig. 3:**
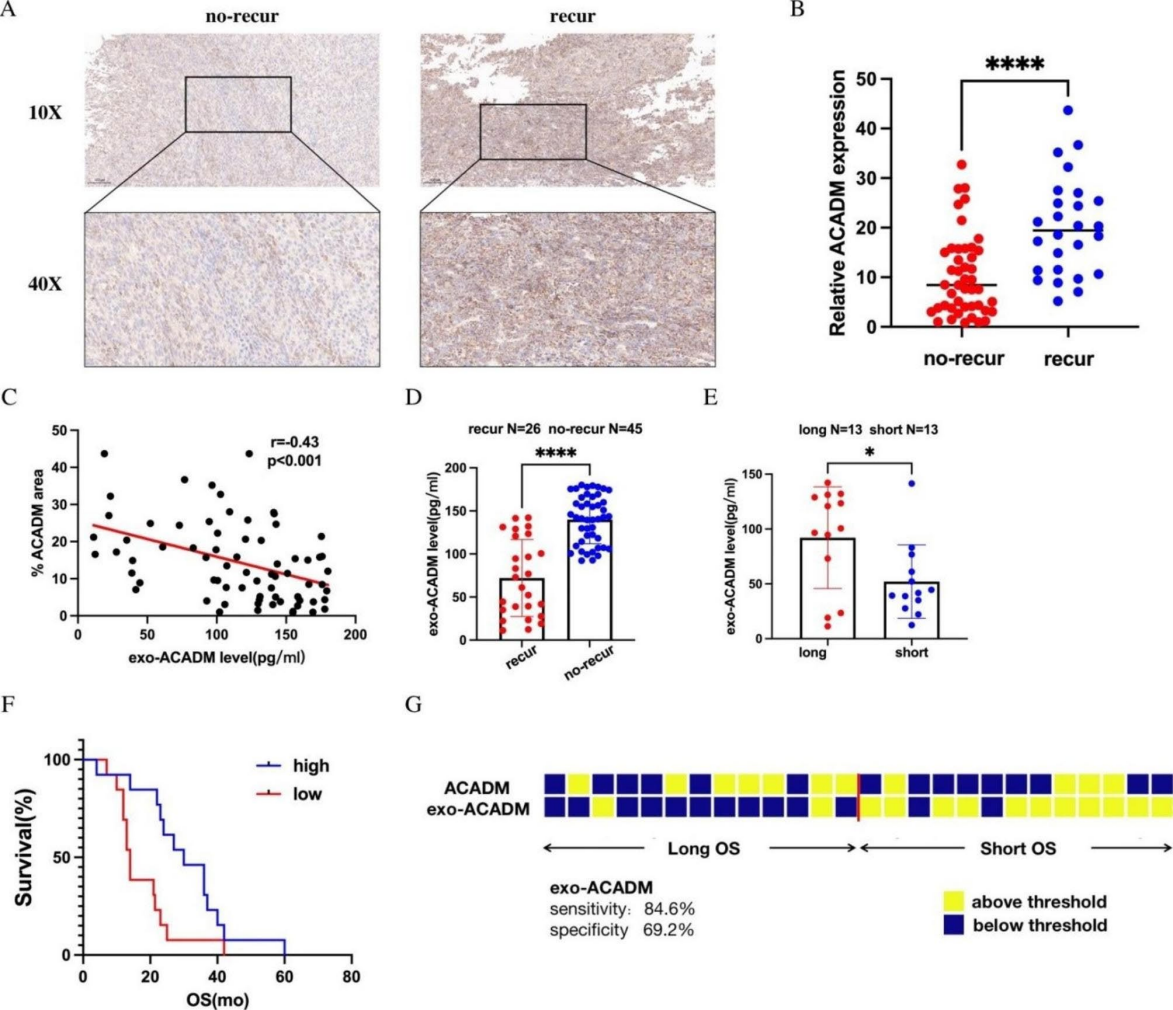
Predictive value of exo-ACADM for chemotherapy relapse. **(A)** Representative IHC staining graph indicated ACADM levels in recurrent and non-recurrent pancreatic cancer. **(B)** Difference in the expression of ACADM between recurrent and non-recurrent pancreatic cancer. **(C)** Correlation analysis between ACADM in tissues and ACADM in patients’ blood exosomes. **(D)** Difference in the expression of exo-ACADM between recurrent and non-recurrent pancreatic cancer. **(E)** Difference in the expression of exo-ACADM between patients with a long and short time of OS **(F)** Survival curves of patients’ recurrent time with high and low expression of exo-ACADM in blood. **(G)** Patterns of high and low values across the exo-ACADM in patients. Each column represents a patient sample

Our experiments demonstrated that exo-ACADM could be used as a reliable indicator of fatty acid metabolism in cells, and an increased expression of exo-ACADM may suggest a reduced response to gemcitabine treatment. To verify this hypothesis, we obtained blood samples from patients who had received gemcitabine chemotherapy after PC resection. Immunohistochemical experiments showed that ACADM levels were significantly higher in patients with tumor recurrence (Fig. 3A and B). This finding was further supported by our in vitro findings (Fig. 3C), which indicated that the level of ACADM in tissues was consistent with that in blood exosomes. Moreover, exo-ACADM expression in the blood of recurrent patients was lower (Fig. 3D). Patients with prolonged overall survival (OS) had higher exo-ACADM expression (Fig. 3E). The Kaplan-Meier analysis revealed that exo-ACADM could differentiate OS (Fig. 3F). Furthermore, exo-ACADM thresholds provided higher sensitivity (84.6%) and specificity (69.2%) than ACADM in tissue (Fig. 3G). Taken together, these results suggest that exosomal ACADM in the blood of patients with PC can predict their postoperative gemcitabine chemosensitivity.

### ACADM regulated chemosensitivity via fatty acid degradation in PC cells

**Fig. 4:**
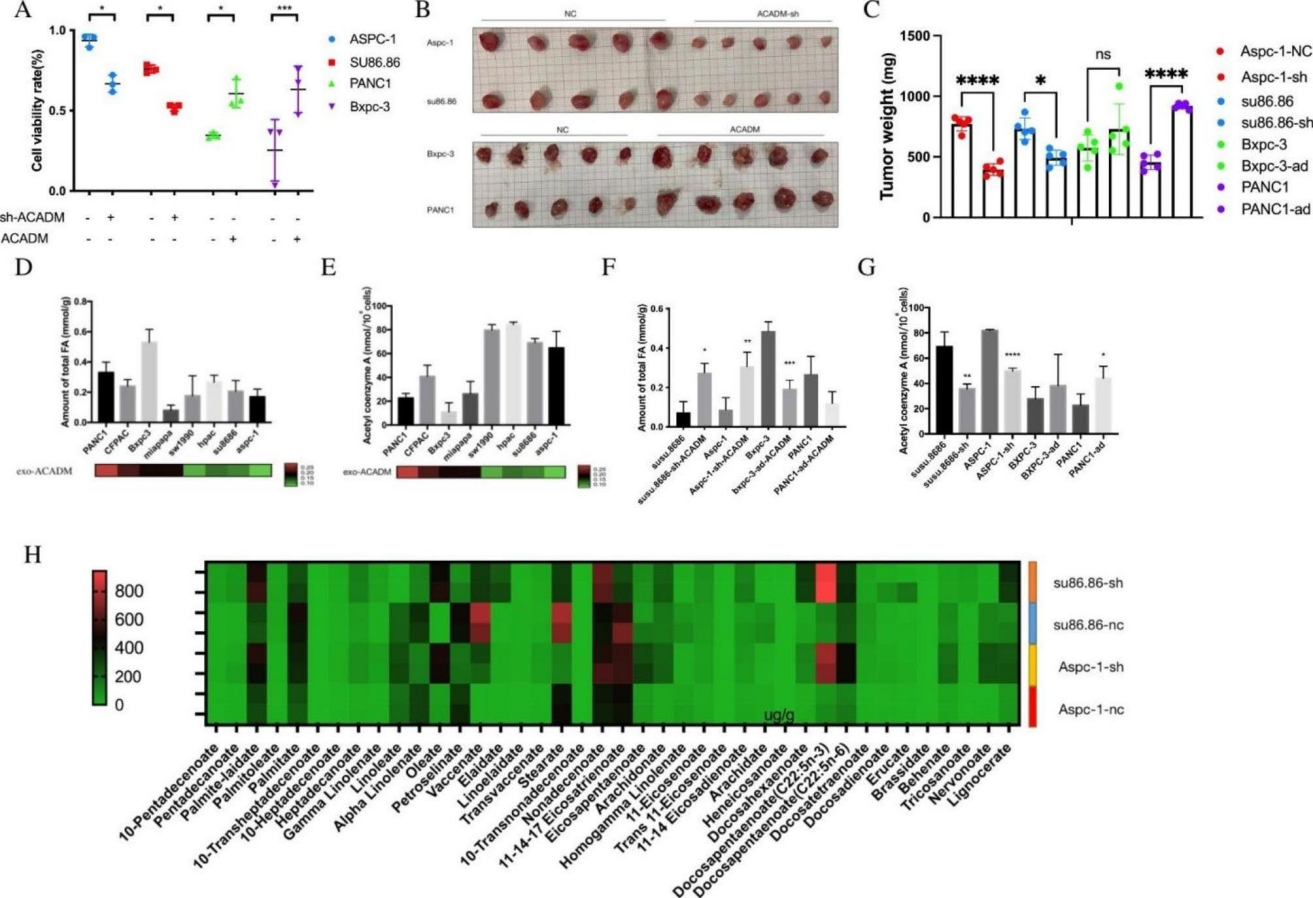
ACADM regulated chemosensitivity via fatty acid degradation in pancreatic cancer cells. **(A)**Activity of pancreatic cancer cells under gemcitabine IC50 concentration (ACADM knockdown or overexpression). **(B)** Xenografts of each cell line (ACADM knockdown or control group) treated with gemcitabine. **(C)**Tumor weight of xenografts treated with gemcitabine. **(D)**The total fatty acid level in PC cells and its relationship with exo-ACADM level. **(E)** The Acetyl-CoA level in PC cells and its relationship with exo-ACADM level. **(F)**The total fatty acid level in PC cells after transfection of ACADM knockdown or overexpression plasmid. **(G)**The Acetyl-CoA level in PC cells after transfection of ACADM knockdown or overexpression plasmid. **(H)** Heatmap showing the level of medium and long-chain fatty acids after ACADM knockdown

Analysis of ACADM in exosomes suggested that it may be a potential indicator of the level of ACADM and sensitivity to gemcitabine in PC cells. To further investigate the effects of ACADM on drug resistance, stable clones of ACADM knockdown and overexpression were generated and verified by Western blot (Figure S1A). Results demonstrated that the knockdown of ACADM increased the sensitivity of PC cells to gemcitabine, while overexpression had the opposite effect (Fig. 4A). Moreover, tumors in the ACADM-knockdown group exhibited a marked decrease in volume and weight after gemcitabine treatment compared to cells in the control group (Fig. 4B C). Additionally, ACADM has been reported to promote hydrolysis of fatty acids, [18] and fatty acid profiling revealed a decrease in fatty acids in exo-ACADM low cells (Fig. 4D). We conducted a study to evaluate the impact of ACADM on fatty acid-related energy metabolism and its correlation to the sensitivity of different cells to gemcitabine. We observed that acetyl-CoA levels were lower in cell lines with high expression of exo-ACADM (Fig. 4E). In the ACADM overexpression group, a significant decrease in fatty acid (FA) was observed in the Bxpc-3 cell line, while in the PANC1 cell line, the FA decreased compared to the control. However, the difference was not statistically significant (Fig. 4F). Furthermore, the knockdown of ACADM reduced acetyl-CoA levels in su86.86 and Aspc-1 cell lines, while overexpression of ACADM significantly increased the level of acetyl-CoA in PANC1 only (Fig. 4G). To further investigate these findings, lipid mass spectrometry was employed to detect the difference between medium- and long-chain fatty acids in drug-resistant cell lines before and after ACADM knockdown (Fig. 4H). The two fatty acids with the most significant difference were palmitoleic acid (POA) and linolenic acid (LA), which increased after ACADM knockdown, while palmitic acid (PA) increased in Aspc-1 cells. Our research suggests that cells with high expression of ACADM can better hydrolyze medium- and long-chain fatty acids, potentially affecting their sensitivity to gemcitabine.

### ACADM knockdown enhanced the FA-triggered cytotoxic effects of gemcitabine by inducing ferroptosis

**Fig. 5:**
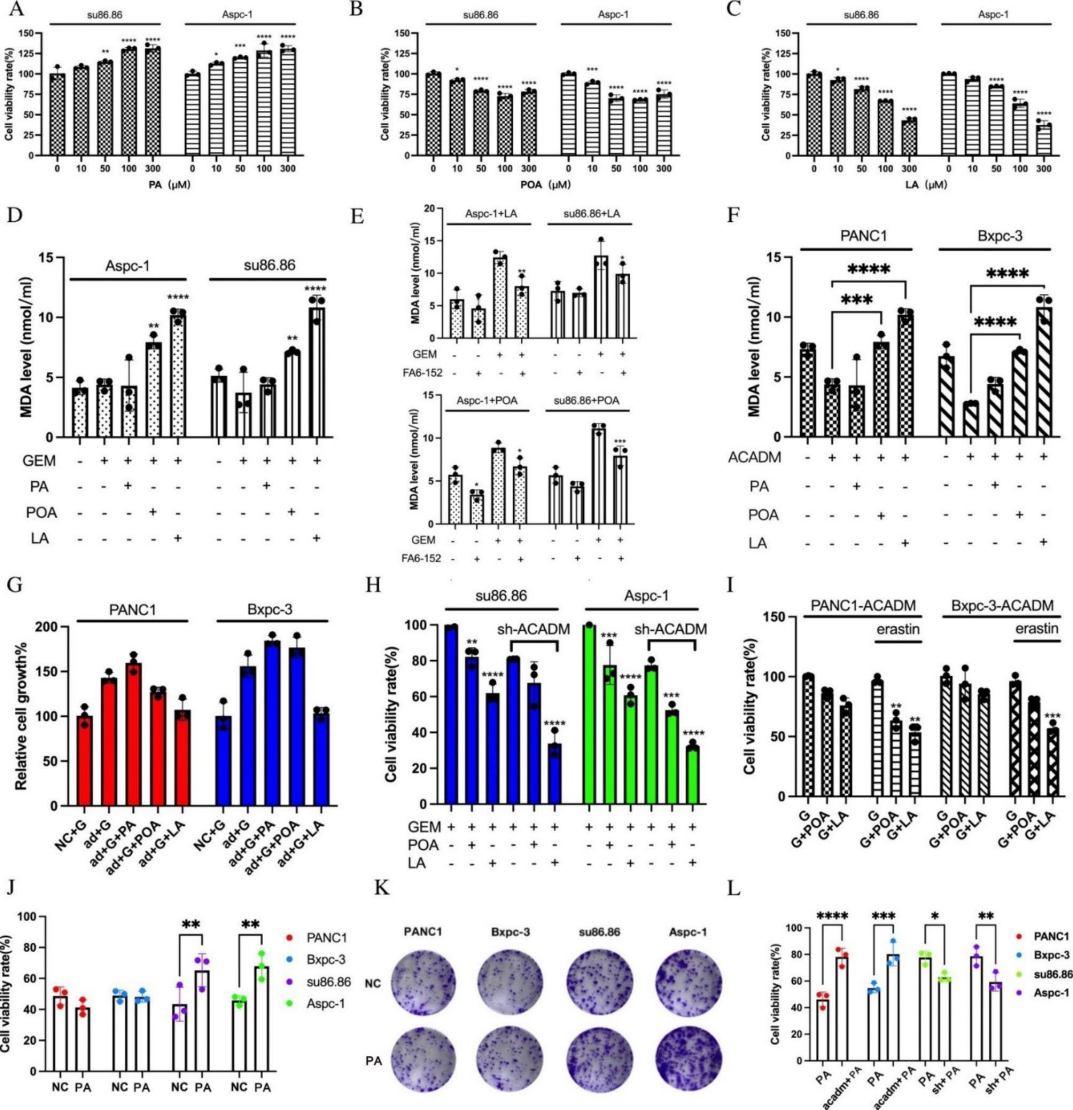
ACADM knockdown enhanced the FA-triggered cytotoxic effects of gemcitabine by inducing ferroptosis. **(A)** Cell viability after treatment with palmitic acid (PA) at different concentrations and gemcitabine. **(B)** Cell viability after treatment with palmitoleic acid (POA) at different concentrations and gemcitabine. **(C)** Cell viability after treatment with linolenic acid (LA) at different concentrations and gemcitabine. **(D)** The MDA level of PC cells after different fatty acid and gemcitabine treatments. **(E)** The MDA level of PC cells after FA6-152 and gemcitabine treatment. **(F)** The MDA level of PC cells with ACADM overexpression. **(G)** ACADM overexpression PC **c**ells viability after treatment with fatty acid. **(H)** ACADM knockdown PC **c**ells viability after treatment with fatty acid. **(I) C**ells viability after treatment with fatty acid and erastin. **(J)** Cell viability of PC cells treated with gemcitabine and palmitic acid (100umol) vs. the untreated control. **(K)** Cloning of pancreatic cancer cells treated with gemcitabine and palmitic acid. **(L)** Cell viability of PC cells treated with gemcitabine and palmitic acid(100umol) of each group

We experimented with assessing the effects of fatty acids and gemcitabine on PC cells with ACADM knockdown. Our results showed that supplementation with POA and LA increased gemcitabine cytotoxicity, while PA had the opposite effect (Fig. 5A C). Additionally, we observed that long-chain unsaturated fatty acids caused lipid peroxidation and cell damage, as evidenced by the increased MDA levels in cells (LA > POA) (Fig. 5D). This effect was reduced when cells were treated with a CD36-blocking antibody (Fig. 5E). Moreover, ACADM overexpression weakened the synergistic effect of unsaturated fatty acids and GEM (Fig. 5F and G), while POA and LA had a more pronounced impact when ACADM was knocked down (Fig. 5H). Furthermore, we found that erastin, a ferroptosis inducer, synergistically affected the cytotoxicity of unsaturated fatty acids (Fig. 5I). In contrast, PA was found to enhance the resistance of PC cells to gemcitabine at 100µM (Fig. 5J K), and this effect was regulated by ACADM (Fig. 5L).

### ACADM prevented peroxidation of PC cells through GPX4

**Fig. 6:**
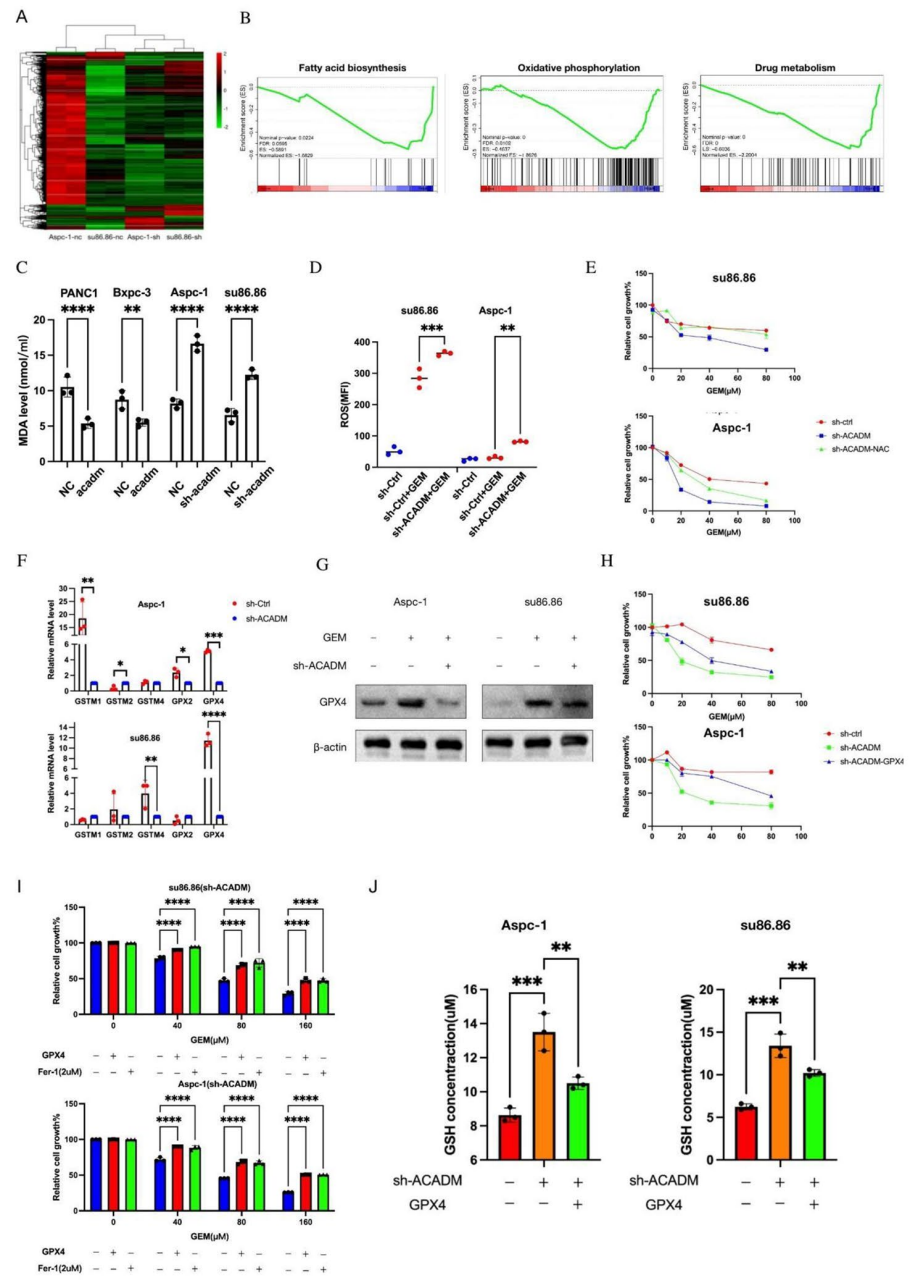
ACADM knockdown enhanced the FA-triggered cytotoxic effects of gemcitabine by inducing ferroptosis **(A)** Heatmap of differential genes after knockdown of ACADM. **(B)** Gene set enrichment analysis of drug metabolism, oxidative phosphorylation, and fatty acid biosynthesis-related genes in ACADM knockdown cell vs. the control group. **(C)** Fatty acid peroxisome level of PC cells after ACADM knockdown or overexpression under gemcitabine treatment. **(D)** ROS level and mean fluorescence intensity (MFI) of pancreatic cancer cells. **(E)** Cell viability of PC cells after treated with or without NAC or knockdown of ACADM. **(F)** The level of glutamine metabolism signature expression was detected in sh-control and sh-ACADM cells by qPCR. **(G)** The GPX4 level of each PC cells were detected by WB. **(H)** Cell viability of PC cells after ACADM knockdown and GPX4 overexpression. **(I)** Cell viability of PC cells in different GEM concentration after treated with Fer-1 or GPX4 overexpression. **(J)** The concentration of glutathione (GSH) was assayed

We performed transcriptome sequencing on ACADM control and knockdown cells to investigate the influence of ACADM and cellular gene expression on gemcitabine sensitivity in PC cells (Fig. 6A). Enrichment analysis showed a decrease in fatty acid synthesis, likely due to product accumulation, as well as weakened intracellular oxidative phosphorylation and drug metabolism (Fig. 6B). Previous studies have suggested that lipid peroxidation can lead to the generation of cytotoxic ROS. However, cells with high ACADM expression were resilient. To assess the effects of ACADM on lipid peroxidation and ROS levels in PC cells, we observed that the knockdown of ACADM decreased peroxidation in AsPC-1 and su86.86, while ACADM overexpression had the reverse effect (Fig. 6C). A ROS probe was used to measure the level of ROS in different cell lines, and the average fluorescence level of ROS decreased upon ACADM knockdown (Fig. 6D). Treatment with the ROS antagonist N-acetylcysteine (NAC) further reduced the sensitivity of cells to chemotherapy (Fig. 6E). To further investigate the role of ROS in regulating ferroptosis, qPCR was conducted to measure the expression of Glutathione metabolism-related enzymes, revealing that GPX4 expression was reduced in ACADM knockdown cell lines (Fig. 6F). Western blot results further confirmed the decreased expression of GPX4 after ACADM knockdown (Fig. 6G). The expression level of GPX4 in the su86 cell line was significantly different after ACADM knockdown, with the difference being more pronounced than in the Aspc-1 cell line. Treatment with the ferroptosis inhibitor Fer-1, or overexpression of GPX4, significantly increased the cell viability of ACADM knockdown cells treated with gemcitabine (Fig. 6H-I). Lastly, the GSH level was measured in the cells, with the results showing that GSH levels increased after ACADM knockdown and were reversed upon GPX4 overexpression (Fig. 6J). Taken together, these findings suggest that the GPX4-mediated high GSH-consumption state is the critical factor of gemcitabine resistance cells via ACADM.

### ACADM upregulated GPX4 via the mevalonate pathway

**Fig. 7:**
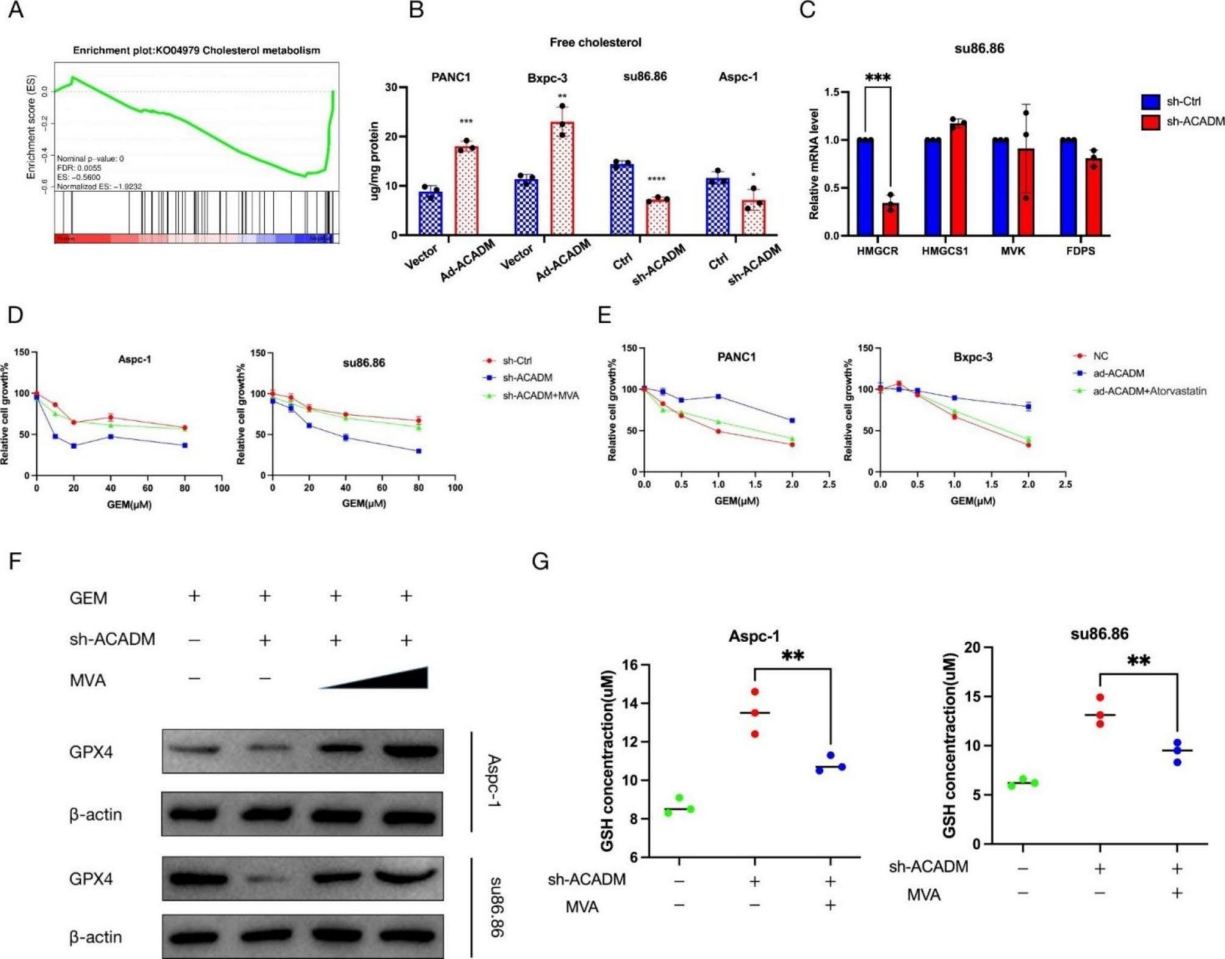
ACADM upregulated GPX4 via mevalonate pathway. **(A)** Gene set enrichment analysis of cholesterol metabolism-related genes in ACADM knockdown cell vs. the control group. **(B)** Free cholesterol level of each PC cells after ACADM knockdown of overexpression. **(C)** The level of MVA-signature expression was detected in sh-control and sh-ACADM cells by qPCR. **(D)** Cell viability of PC cells in three group (GEM + sh-Ctrl,GEM + sh-ACADM, GEM + sh-ACADM + MVA). **(E)** Cell viability of PC cells in three group (GEM + ad-Ctrl,GEM + ad-ACADM, GEM + ad-ACADM + Atorvastatin). **(F)** The GPX4 level of each PC cells were detected by WB. **(G)** The concentration of glutathione (GSH) was assayed

Our transcriptome sequencing results indicated that the activity of cholesterol metabolism was inhibited in the ACADM knockdown group compared to the control group (Fig. 7A). This pathway is essential for the GPX4 synthesis of tRNA. We observed that the level of free cholesterol decreased in Aspc-1 and su86.86 cells after ACADM knockdown, while the opposite was seen in PANC-1 and Bxpc-3 overexpression (Fig. 7B). Additionally, qPCR analysis of mevalonate pathway-related genes such as HMGCR, HMGCS1, and MVK showed that HMGCR expression was reduced in ACADM knockdown cells (Fig. 7C, Figure S1B). Furthermore, exogenous MVA supplementation reversed the sensitivity of PC cells to gemcitabine after ACADM knockdown in the CCK-8 experiment. In sensitive cell lines overexpressing ACADM, inhibition of cholesterol synthesis (atorvastatin) restored the cytotoxicity of gemcitabine (Fig. 7D-E). Lastly, our findings were further validated by detecting the level of GPX4 and GSH concentration in cells after ACADM knockdown in the presence of exogenous MVA supplementation, demonstrating that ACADM regulates GPX4 expression in an MVA pathway-dependent manner (Fig. 7F-G).

## Discussion

This study sought to evaluate the efficacy of exo-ACADM as a biomarker to determine a patient’s sensitivity to gemcitabine chemotherapy in PC. Obtaining tissue samples from PC can be challenging, thus necessitating the use of blood tests to predict a patient’s responsiveness to chemotherapy. Postoperative chemotherapy resistance significantly reduces survival time, and a reliable pre-operative assessment can avoid ineffective surgery and provide a more tailored postoperative plan. Moreover, those with metastatic cancer who are ineligible for surgery can benefit from the guidance of blood tests in formulating neoadjuvant treatment. The predictive capability of exosomes from cell lines with varying sensitivities to gemcitabine was explored and validated using xenografts and clinical samples.

Experiments have demonstrated that exosomes can mediate cell-to-cell communication and transfer noncoding RNA [19, 20]. Additionally, research has been conducted to examine the modifications in tumor cell-derived exosomes and their effects on the tumor microenvironment post-chemotherapy. For instance, when myeloma cells were exposed to chemo-exosomes, the heparanase cargo was transferred, resulting in increased heparan sulfate degrading activity, ERK signaling activation, and increased shedding of syndecan-1 proteoglycan [21]. To predict chemosensitivity, exosome components are typically examined after treatment with chemotherapeutic drugs [22]. In this study, we utilized mass spectrometry and sequencing technology to investigate the molecular and ACADM variations in the secretion of various sensitive PC cells and identify predictive markers of sensitivity to gemcitabine chemotherapy. This method could benefit the pre-operative assessment of treatment efficacy, thus avoiding unnecessary surgical interventions and devising more effective treatment regimens. Our results were further validated in a retrospective study on clinical PC samples, which showed that exo-ACADM had superior predictive capacity than ACADM in tissue. To further evaluate the clinical value of exo-ACADM, prospective studies need to be conducted.

Research has illustrated that cancer cells profoundly influence fatty acid metabolism. For example, FABP5 can manage fatty acid absorption, and its higher expression on ER and PR receptor-negative breast cancer cells is linked to a poor prognosis [23, 24]. CD36 is highly expressed in gliomas and has been related to tumor progression [25]. A study has revealed that heightening the FAO pathway may facilitate lung cancer progression and osimertinib resistance. ACADM, an essential gene in the first step of fatty acid oxidation and degradation of medium- and long-chain fatty acids, can lead to significant mortality and morbidity rates in undiagnosed patients. Thus, potential adverse effects can be prevented by simply reducing metabolic stress and taking dietary precautions [26]. However, the effects of ACADM on cancer have been scarcely studied. Yam’s research showed that when ACADM-mediated fatty acid β-oxidation is blocked, it can encourage the emergence of liver cancer [18]. Studies have demonstrated that cancer cells can synthesize and absorb more significant amounts of fatty acids, including palmitic acid. It has been proposed that preventing the accumulation of palmitic acid may stimulate tumor cell apoptosis [27]. In this study, an analysis of cell lines with various levels of gemcitabine sensitivity exposed that drug-resistant cells could augment fatty acid oxidation (FAO) and utilize medium- and long-chain fatty acids. It is known that excessive FAO generates reactive oxygen species (ROS), which can cause cell apoptosis [28–30]. However, our results demonstrated that drug-resistant cells could manage ROS production induced by FAO and avoid apoptosis.

Our research demonstrated that ACADM could catalyze the oxidation of fatty acids. However, drug-resistant cells can reduce reactive oxygen species (ROS) levels to avoid cell death, as evidenced by malondialdehyde (MDA) and ROS detection results. The antioxidant enzyme GPX4 can reduce peroxide to non-toxic alcohol through glutamine metabolism [16, 31]. We observed that the level of GPX4 decreased, and the glutathione (GSH) concentration increased after ACADM was knocked down. Intriguingly, the ROS antagonist NAC could reverse the cytotoxic effects of gemcitabine after ACADM knockdown. Thus, it is essential to maintain a balance between antioxidant reactions and ROS production to ensure the chemosensitivity of tumor cells.

The effects of ACADM on GPX4 expression were further examined. It was observed that the rate of cholesterol synthesis decreased when ACADM was inhibited, likely due to the decrease in acetyl-CoA caused by the inhibition of fatty acid metabolism [32]. Statins, which are mevalonate pathway inhibitors, have been used in clinical practice, yet their efficacy in increasing chemosensitivity is still uncertain. Ji et al. revealed the role of the MVA-GGPP metabolic pathway in chemotherapy-resistant small-cell lung cancer and proposed that statins targeting this pathway could be a potential strategy to combat resistance in clinical treatments [33]. Our findings showed that drug-resistant cells with high ACADM expression were more responsive to gemcitabine when treated with statins. To validate these results, further research should be conducted using xenograft tumors and patient-derived tumor xenografts (PDXs).

In conclusion, exo-ACADM could reflect fatty acid metabolism and gemcitabine sensitivity in PC, potentially providing a reliable measure of the efficacy of chemotherapy treatments.

### Electronic supplementary material

Below is the link to the electronic supplementary material.

Supplementary Material 1

Supplementary Material 2

## Data Availability

The datasets generated and/or analysed during the current study are available in the SRA repository (PRJNA942205).

## References

[1] Kleeff J, Korc M, Apte M, La Vecchia C, Johnson CD, Biankin AV, Neale RE, Tempero M, Tuveson DA, Hruban RH (2016). Pancreatic cancer. Nat Rev Dis Primers.

[2] Sarvepalli D, Rashid MU, Rahman AU, Ullah W, Hussain I, Hasan B, Jehanzeb S, Khan AK, Jain AG, Khetpal N (2019). Gemcitabine: a review of Chemoresistance in Pancreatic Cancer. Crit Rev Oncog.

[3] Binenbaum Y, Na’ara S, Gil Z (2015). Gemcitabine resistance in pancreatic ductal adenocarcinoma. Drug Resist Updat.

[4] Gao C, Wisniewski L, Liu Y, Staal B, Beddows I, Plenker D, Aldakkak M, Hall J, Barnett D, Gouda MK (2021). Detection of chemotherapy-resistant pancreatic Cancer using a glycan biomarker, sTRA. Clin Cancer Res.

[5] Ham S, Lima LG, Chai EPZ, Muller A, Lobb RJ, Krumeich S, Wen SW, Wiegmans AP, Möller A (2018). Breast Cancer-derived Exosomes alter macrophage polarization via gp130/STAT3 signaling. Front Immunol.

[6] Wang X, Luo G, Zhang K, Cao J, Huang C, Jiang T, Liu B, Su L, Qiu Z (2018). Hypoxic tumor-derived exosomal miR-301a mediates M2 macrophage polarization via PTEN/PI3K gamma to promote pancreatic Cancer metastasis. Cancer Res.

[7] Yang Q, Zhao S, Shi Z, Cao L, Liu J, Pan T, Zhou D, Zhang J (2021). Chemotherapy-elicited exosomal miR-378a-3p and miR-378d promote breast cancer stemness and chemoresistance via the activation of EZH2/STAT3 signaling. J Exp Clin Cancer Res.

[8] Lan B, Zeng S, Grützmann R, Pilarsky C (2019). The role of Exosomes in Pancreatic Cancer. Int J Mol Sci.

[9] Zhang Y, Bi J, Huang J, Tang Y, Du S, Li P (2020). Exosome: a review of its classification, isolation techniques, Storage, Diagnostic and targeted therapy applications. Int J Nanomedicine.

[10] Al-Bahlani S, Al-Lawati H, Al-Adawi M, Al-Abri N, Al-Dhahli B, Al-Adawi K (2017). Fatty acid synthase regulates the chemosensitivity of breast cancer cells to cisplatin-induced apoptosis. Apoptosis.

[11] Rios Garcia M, Steinbauer B, Srivastava K, Singhal M, Mattijssen F, Maida A, Christian S, Hess-Stumpp H, Augustin HG, Müller-Decker K (2017). Acetyl-CoA carboxylase 1-Dependent protein acetylation controls breast Cancer metastasis and recurrence. Cell Metab.

[12] Lee JY, Kim WK, Bae KH, Lee SC, Lee EW (2021). Lipid metabolism and ferroptosis. Biology (Basel).

[13] Golbashirzadeh M, Heidari HR, Talebi M, Yari Khosroushahi A (2023). Ferroptosis as a potential cell death mechanism against cisplatin-resistant Lung Cancer Cell line. Adv Pharm Bull.

[14] You JH, Lee J, Roh JL (2021). Mitochondrial pyruvate carrier 1 regulates ferroptosis in drug-tolerant persister head and neck cancer cells via epithelial-mesenchymal transition. Cancer Lett.

[15] Zhang X, Ma Y, Ma J, Yang L, Song Q, Wang H, Lv G (2022). Glutathione peroxidase 4 as a therapeutic target for Anti-Colorectal Cancer Drug-Tolerant Persister cells. Front Oncol.

[16] Xie Y, Hou W, Song X, Yu Y, Huang J, Sun X, Kang R, Tang D (2016). Ferroptosis: process and function. Cell Death Differ.

[17] Kanehisa M, Goto S (2000). KEGG: Kyoto Encyclopedia of genes and genomes. Nucleic Acids Res.

[18] Ma APY, Yeung CLS, Tey SK, Mao X, Wong SWK, Ng TH, Ko FCF, Kwong EML, Tang AHN, Ng IO (2021). Suppression of ACADM-Mediated fatty acid oxidation promotes Hepatocellular Carcinoma via aberrant CAV1/SREBP1 signaling. Cancer Res.

[19] Lai JJ, Chau ZL, Chen SY, Hill JJ, Korpany KV, Liang NW, Lin LH, Lin YH, Liu JK, Liu YC (2022). Exosome Processing and characterization approaches for Research and Technology Development. Adv Sci (Weinh).

[20] Skotland T, Hessvik NP, Sandvig K, Llorente A (2019). Exosomal lipid composition and the role of ether lipids and phosphoinositides in exosome biology. J Lipid Res.

[21] Bandari SK, Purushothaman A, Ramani VC, Brinkley GJ, Chandrashekar DS, Varambally S, Mobley JA, Zhang Y, Brown EE, Vlodavsky I (2018). Chemotherapy induces secretion of exosomes loaded with heparanase that degrades extracellular matrix and impacts tumor and host cell behavior. Matrix Biol.

[22] Lin Y, Dong H, Deng W, Lin W, Li K, Xiong X, Guo Y, Zhou F, Ma C, Chen Y (2019). Evaluation of salivary exosomal chimeric GOLM1-NAA35 RNA as a potential biomarker in esophageal carcinoma. Clin Cancer Res.

[23] Liu RZ, Graham K, Glubrecht DD, Germain DR, Mackey JR, Godbout R (2011). Association of FABP5 expression with poor survival in triple-negative breast cancer: implication for retinoic acid therapy. Am J Pathol.

[24] Zhang C, Liao Y, Liu P, Du Q, Liang Y, Ooi S, Qin S, He S, Yao S, Wang W (2020). FABP5 promotes lymph node metastasis in cervical cancer by reprogramming fatty acid metabolism. Theranostics.

[25] Hale JS, Otvos B, Sinyuk M, Alvarado AG, Hitomi M, Stoltz K, Wu Q, Flavahan W, Levison B, Johansen ML (2014). Cancer stem cell-specific scavenger receptor CD36 drives glioblastoma progression. Stem Cells.

[26] Maier EM, Liebl B, Röschinger W, Nennstiel-Ratzel U, Fingerhut R, Olgemöller B, Busch U, Krone N, v Kries R, Roscher AA (2005). Population spectrum of ACADM genotypes correlated to biochemical phenotypes in newborn screening for medium-chain acyl-CoA dehydrogenase deficiency. Hum Mutat.

[27] Fatima S, Hu X, Gong RH, Huang C, Chen M, Wong HLX, Bian Z, Kwan HY (2019). Palmitic acid is an intracellular signaling molecule involved in disease development. Cell Mol Life Sci.

[28] Li XX, Wang ZJ, Zheng Y, Guan YF, Yang PB, Chen X, Peng C, He JP, Ai YL, Wu SF (2018). Nuclear receptor Nur77 facilitates Melanoma Cell Survival under metabolic stress by protecting fatty acid oxidation. Mol Cell.

[29] Chen X, Li J, Kang R, Klionsky DJ, Tang D (2021). Ferroptosis: machinery and regulation. Autophagy.

[30] Curi R, Levada-Pires AC, Silva EBD, Poma SO, Zambonatto RF, Domenech P, Almeida MM, Gritte RB, Souza-Siqueira T, Gorjão R (2020). The critical role of cell metabolism for essential neutrophil functions. Cell Physiol Biochem.

[31] Forcina GC, Dixon SJ (2019). GPX4 at the crossroads of lipid homeostasis and ferroptosis. Proteomics.

[32] Juarez D, Fruman DA (2021). Targeting the Mevalonate Pathway in Cancer. Trends Cancer.

[33] Guo C, Wan R, He Y, Lin SH, Cao J, Qiu Y, Zhang T, Zhao Q, Niu Y, Jin Y (2022). Therapeutic targeting of the mevalonate-geranylgeranyl diphosphate pathway with statins overcomes chemotherapy resistance in small cell lung cancer. Nat Cancer.

