# Supplementary material for: Exosomal ACADM sensitizes gemcitabine-resistance through modulating fatty acid metabolism and ferroptosis in pancreatic cancer
Source: BMC Cancer. 2023 Aug 23;23:789. doi: 10.1186/s12885-023-11239-w (PMC10463774; doi:10.1186/s12885-023-11239-w)
Supplement: Supplementary file 1 — Supplementary Material 1 [file 12885_2023_11239_MOESM1_ESM.pdf]

Supplement Figure

A

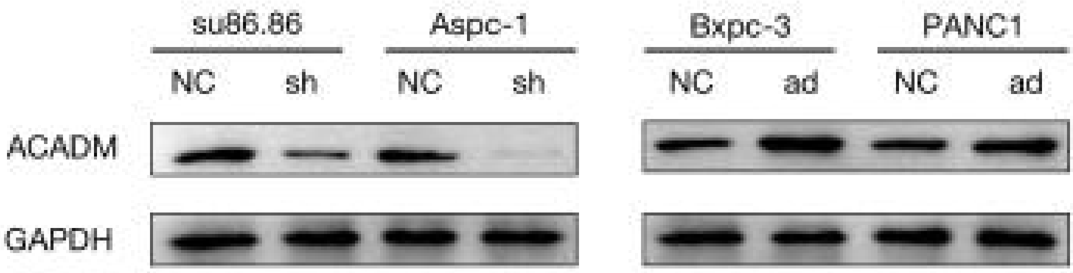

B

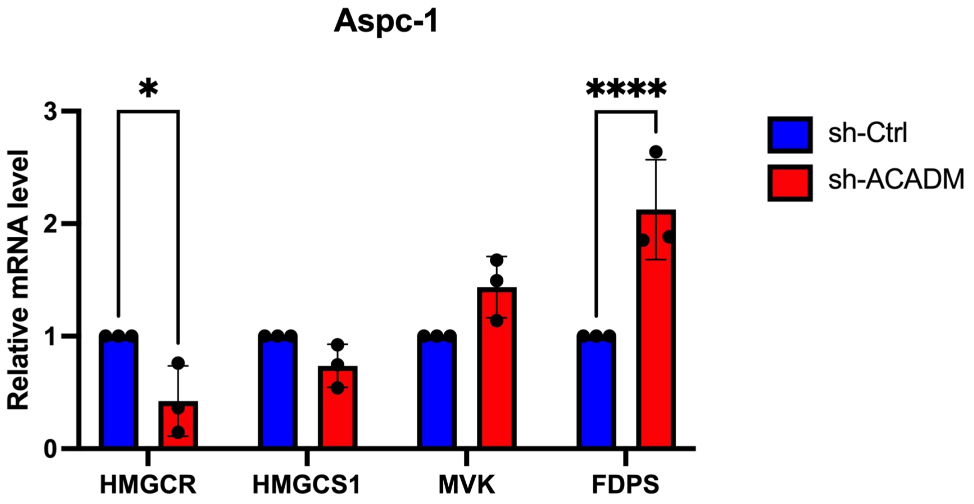

Figure legend

(A) Western blot showed the level of ACADM in pancreatic cancer cells after ACADM knockdown and overexpression.

(B) The level of MVA-signature expression were detected in sh-control and sh-ACADM cells by qPCR.
