# Supplementary material for: Exosomal ACADM sensitizes gemcitabine-resistance through modulating fatty acid metabolism and ferroptosis in pancreatic cancer
Source: BMC Cancer. 2023 Aug 23;23:789. doi: 10.1186/s12885-023-11239-w (PMC10463774; doi:10.1186/s12885-023-11239-w)

Supplement Figure1：Uncropped gel images of western blot

Figure1D

CD63

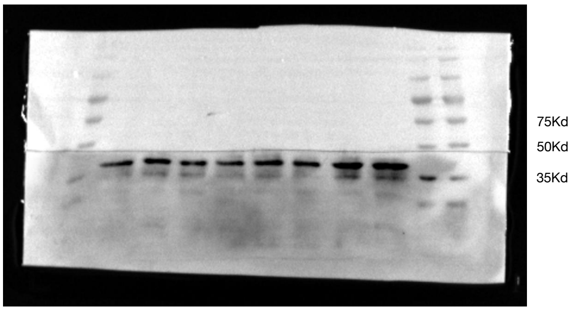

CD81

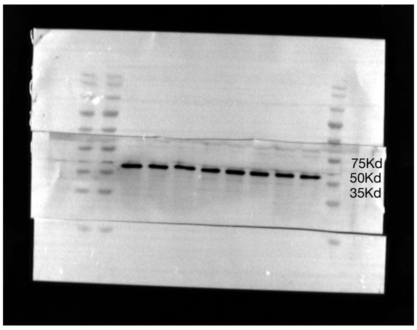

Figure2B and 2C

ACADM

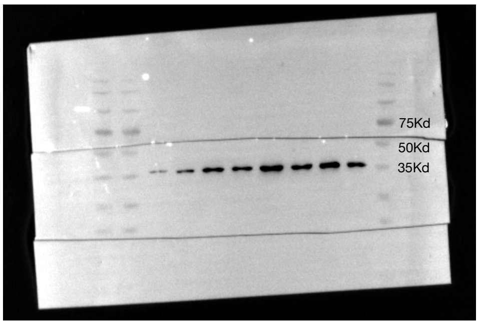

CD63

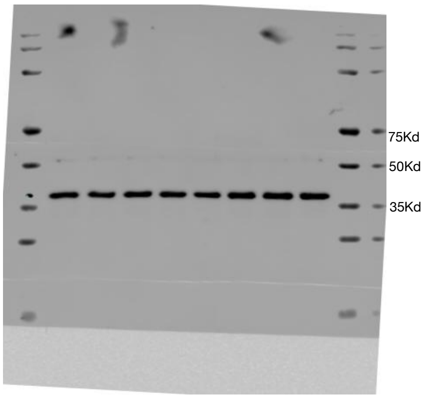

Figure2D and 2E

ACADM

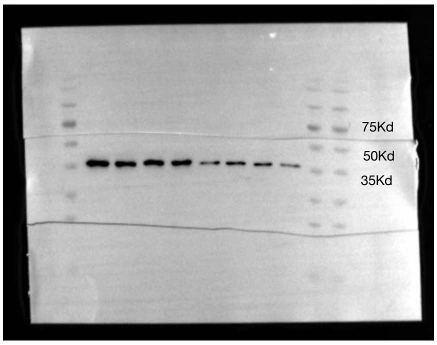

β -actin

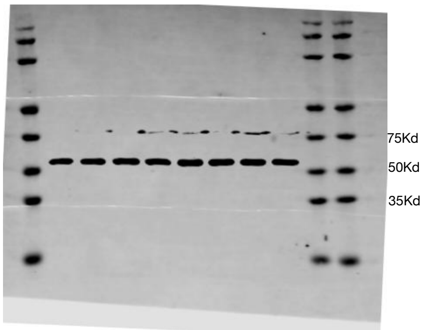

Figure5G

GPX4

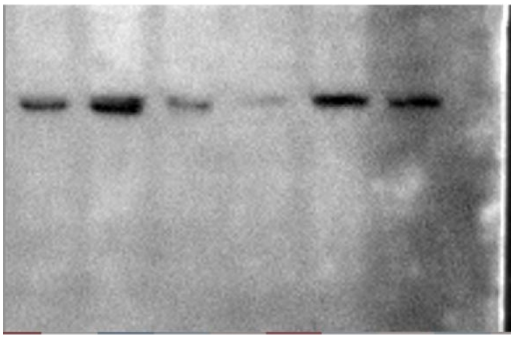

β -actin

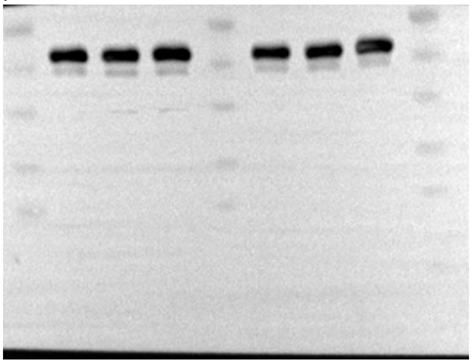

Figure7F

GPX4

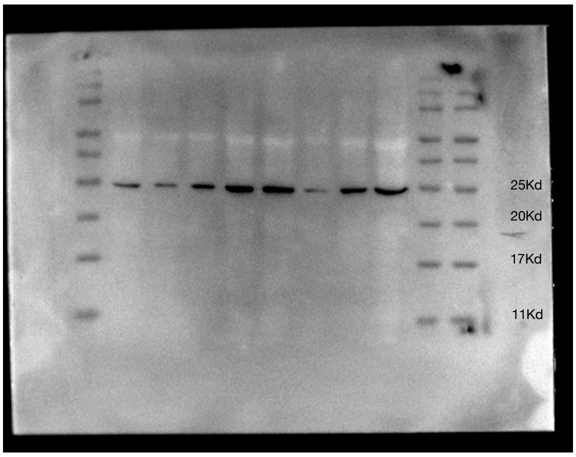

β actin

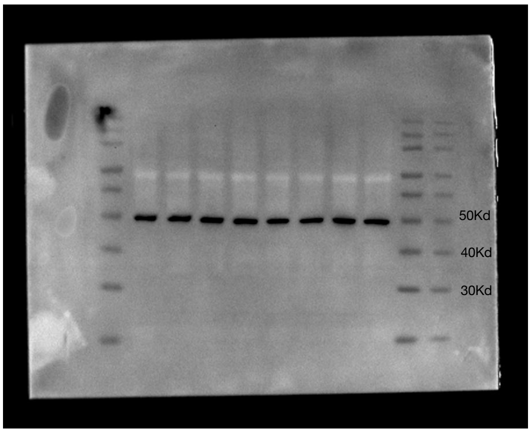

Supplement: Supplementary file 2 — Supplementary Material 2 [file 12885_2023_11239_MOESM2_ESM.pdf]
